# Environmental and non-infectious factors in the aetiology of pharyngitis (sore throat)

**DOI:** 10.1007/s00011-012-0540-9

**Published:** 2012-08-14

**Authors:** Bertold Renner, Christian A. Mueller, Adrian Shephard

**Affiliations:** 1 Department of Experimental and Clinical Pharmacology, University of Erlangen-Nuremberg, Krankenhausstr. 9, 91054 Erlangen, Germany; 2 Department of Otorhinolaryngology, Medical University of Vienna, Vienna, Austria; 3 Reckitt Benckiser Group plc, Slough, UK

**Keywords:** Air pollution, Cold dry air, Experimental model, Inflammation, Occupational exposure, Therapy, Pain

## Abstract

**Objectives:**

The aim of this review is to examine the causes, pathophysiology and experimental models of non-infectious pharyngitis (sore throat).

**Introduction:**

The causes of sore throat can be infectious (viruses, bacteria, and fungi) or non-infectious, although the relative proportion of each is not well documented.

**Methods:**

A PubMed database search was performed for studies of non-infectious sore throat.

**Results and conclusions:**

Non-infectious causes of sore throat include: physico-chemical factors, such as smoking, snoring, shouting, tracheal intubation, medications, or concomitant illness; and environmental factors including indoor and outdoor air pollutants, temperature and humidity, and hazardous or occupational irritants. The pathophysiology underlying non-infectious sore throat is largely uncharacterised, although neurogenic inflammation looks to be a promising candidate. It is likely that there will be individual disposition factors or the coincidence of more than one irritant with possible—up to now unknown—interactions between them. Therefore, experimental models with defined conditions and objective endpoints are needed. A new model using cold dry air to directly induce pharyngeal irritation in humans, with pharyngeal lavage to measure biomarkers, may provide a useful tool for the study of mechanisms and treatment of non-infectious sore throat.

## Introduction

Pharyngitis is inflammation of the oropharynx. It is commonly referred to as sore throat, although this term is often used imprecisely and is poorly defined. Hence, whilst sore throat may be the symptom described by the patient, examination might also reveal nasopharyngitis (that is, including the nasopharyngeal mucosa). The exact site of the irritation/inflammation is often not identified, with some studies relying on a patient-reported diagnosis (that is, ‘sore throat’). These factors potentially complicate the study (and treatment) of sore throat. The following review will focus on sore throat as the primary symptom, which implies reported irritation and pain caused by inflammation and/or irritation of the oro (medium part) and hypopharynx (lower part of the pharynx). As mentioned, there exist also overlapping symptoms to nasopharyngitis (upper part of the pharynx) but rhinitis or rhinosinusitis are usually diagnosed in those patients and sore throat is not the primary symptom.

Sore throat may have an infectious or non-infectious aetiology, although these sometimes overlap. Most cases are infectious, with a large proportion (up to 40 %) caused by rhinovirus and adenovirus. Other viruses including coronavirus, influenza, parainfluenza, Epstein Barr, and herpes simplex have also been implicated [1]. Of the bacterial sore throats, group A β-haemolytic streptococcus is most frequently (5–36 %) isolated [1, 2]. Other organisms to which sore throat has been attributed include *Mycoplasma pneumonia* and *Arcanobacterium haemolyticus* [1]. Rarely, candidal infections and other fungi and parasites have also been observed [1].

**Fig. 1:**
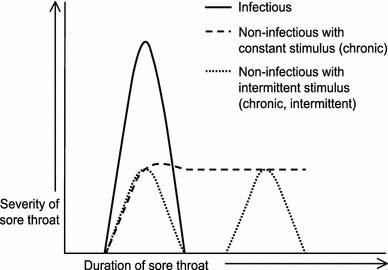
Schematic representation of typical course of sore throat of infectious and non-infectious aetiology

A proportion of sore throats have non-infectious aetiologies, although the relative prevalence versus infectious cases is not well documented. This is probably because it is a costly and difficult area to study. The non-infectious causes of sore throat are extremely varied, and include physico-chemical factors (for example smoking, snoring, shouting, drugs) and environmental factors (for example pollution, temperature, humidity/air conditioning). An approximation might be obtained by identifying people with sore throat in the absence of any other symptom (e.g. rhinosinusitis) or with persistent sore throat (Fig. 1); but this does not definitively exclude a viral, bacterial, or fungal cause. In fact, quantifying the prevalence of non-infectious sore throat would likely require the active identification and exclusion of all potential infectious causes.

Overall, sore throat is a very common complaint and a frequent reason for seeking medical care [3]. Of 15,788 respondents (aged 14 years or older) in a Scottish postal survey, 31 % reported they had experienced severe sore throat or tonsillitis in the previous year [4]. In the USA, acute pharyngitis accounts for 1–2 % of all visits to outpatient departments, physician offices, and emergency departments [5]. Many people do not seek medical care when they have a sore throat [6], although geographical variation is considerable, dependent on social factors, access to and cost of healthcare, and attitudes to antibiotic prescribing.

When patients with sore throat do present to primary care, the optimal management is controversial. This is because, relatively rarely, sore throat can be serious [7]. Furthermore, whilst antibiotics may provide only modest absolute benefits [8], there are few other prescription options available for sore throat. Recurrent severe episodes of sore throat might indicate tonsillectomy, while concurrent severe symptoms such as difficulty breathing or swallowing usually require hospital admission [2]. The main cause for concern though is the risk of rheumatic fever and suppurative complications in patients with group A β-haemolytic streptococcus and for this reason many doctors, particularly in the USA, exercise caution [9]. Other potential complications include post-viral olfactory dysfunction [10] and anosmia (which might be prevented by vaccination in the future). A review of guidelines found that recommended management strategies vary widely [11]. In some countries sore throat is considered to be self-limiting and swabbing and antibiotics are not routine, whereas, others recommend microbiological investigation with subsequent antibiotics for confirmed streptococcal cases [11]. Non-steroidal anti-inflammatory drugs and paracetamol are recommended for symptomatic relief [2, 12], while the relative risks and benefits of corticosteroids require further study [2, 13]. The management of sore throat is, therefore, far from standardised or evidence based.

It is clear then that the majority of people with sore throat have a benign self-limiting illness and many do not present for medical care [6]. Amongst these are a largely unquantified population with non-infectious aetiologies, about which little is understood and for whom the optimal treatment strategy is unclear. The objective of this review was to collate the information on non-infectious sore throat, particularly the proposed aetiologies and mechanisms involved.

## Causes of non-infectious sore throat

### Physico-chemical factors

A wide variety of physico-chemical factors have been implicated in causing sore throat, including cigarette smoke inhalation, snoring, tracheal intubation, shouting, and concomitant illness or drug effects.

*Smoking* is a risk factor for sore throat [14], in both smokers themselves and in those exposed to secondary smoke (passive smoking). Cigarette smoking was significantly associated with sore throat/cough in US college students [15], and frequency of cigarette smoking and sore throat were correlated in Japanese women [16]. Passive smoking was significantly associated with sore throat in 46 French non-smokers [17], 382 Australian non-smoking indoor workers [18], and non-smoking Australian nightclub and casino workers [19]. Furthermore, a questionnaire study reported a significant relationship between children’s sore throats and maternal smoking [20].

*Snoring* is frequently associated with sore throat, and the two have risk factors such as smoking [21] in common, although the direction of causality is not always clear. A high frequency of sore throat is a risk factor for habitual snoring in children [22] and sore throat was reported by over half of those snoring children who were subsequently diagnosed with obstructive sleep apnoea [23]. Sore throat may also be associated with obstructive sleep apnoea in adults [24]. Sleep apnoea is a key factor for the manifestation of secondary hypertension.

*Tracheal intubation* and laryngeal mask airways are common causes of sore throat in people undergoing general anaesthesia [24–27]. The reported incidence of postoperative sore throat varies widely, but is generally higher for tracheal intubation than for laryngeal mask airway [28]. For patients undergoing tracheal intubation, an incidence of around 28–45.5 % [27, 29]— and as high as 70 % [30]— has been reported. For laryngeal mask airway, the incidence is lower—in the region of 3.5–21.4 % [27, 29, 31].

*Shouting* and voice loading may cause sore throat, as reported by people in professions that require use (and overuse) of their voice for their work. For example, aerobics instructors have reported an increased incidence of sore throat unrelated to illness since beginning instructing [32] and the frequency of aerobics classes has been shown to significantly correlate with sore throat symptoms in instructors [33]. Sore throat is also reported by school teachers [34]. Furthermore, sore throat can occur as a secondary consequence of functional dysphonia [35] as well as vice versa.

*Drug-induced* sore throat is a notable adverse effect of some medications, including angiotensin-converting enzyme (ACE) inhibitors [36] and chemotherapy agents. Sore throat is also a common problem in asthmatics taking inhaled corticosteroids [37–39], although these data may be in part confounded by a potential steroid-induced increased susceptibility to infection, including mycoses. In fact, people taking a wide variety of other drugs frequently report sore throat as an adverse effect, but in many cases the symptom will be coincidental (that is, probably infectious sore throat and at a similar rate to placebo).

*Concomitant illness* can directly result in sore throat. For example, Kawasaki disease (a mucocutaneous vasculitis) typically causes pharyngitis in both adults and children [40]. Furthermore, chronic pharyngitis is a common manifestation of gastroesophageal reflux disorder [41], with this presentation now termed laryngopharyngeal reflux disorder [42]. While in these cases the sore throat can be considered to be a direct manifestation of the underlying condition, other illnesses may be associated with sore throat due to a consequent increased predisposition to infection. This may be the case for the often cited link with thyroid disease, where 41 % of patients with newly diagnosed thyroiditis report sore throat as a first symptom [43].

## Environmental factors

A wide variety of environmental factors have been cited as causes of sore throat, including general air pollution, specific pollution related to occupation, hazard, or industry, and pollution encountered in indoor environments. However, because the data are often epidemiological and individual pollutants are rarely emitted in isolation, it is not always possible to identify the precise cause of symptoms. Patients with allergic rhinitis [44] as well as those with postnasal drip [7], non-allergic rhinitis, and persistent cough may also experience sore throat as a secondary symptom.

*Ambient air pollution* is a common cause of sore throat. The factors implicated include ozone, nitrogen oxides, and fine dust.

Urban living and traffic fumes are frequently associated with sore throat. In children in Hong Kong, sore throat decreased when fuel sulphur levels were reduced [45], whilst exposure to vehicle emissions was linked with throat pain and chronic pharyngitis in Shanghai bus drivers/conductors/taxi drivers [46] and lack of air conditioning with frequent sore throat in Hong Kong bus and tram drivers [47]. Nitrogen dioxide has also been implicated [48, 49], as well as photochemical oxidants such as ozone (O_3_) [50, 51].

*Occupational or hazard-associated irritants* that have been reported to cause sore throat include particulates, fumes, chemicals, and odours. Laryngitis has also been reported [52].

Particulates and fumes from a wide variety of industries have been implicated in sore throat, including pulp mills [53], woodworking [54], cement works [55], brick kilns [56], and factory exhaust emissions [57]. The burning of gas creates multiple pollutants, including nitrogen oxides, particulates, and volatile organic compounds, and gas-related pollution has been linked with sore throat in several studies [58, 59]. Cooking and fires are important sources of pollutants worldwide [60], and in US non-smoking women, each hour of fireplace use was associated with (little but measurable) increased episodes of sore throat (relative risk 1.04; 95 % CI 1.00–1.08) [61].

A variety of chemicals have been reported to cause sore throat including organic screen-printing solvents [62], boron oxide, boron acid, and borax dust [63, 64], fluorinated hydrocarbons [65], machining coolants in the metal working industry [66], nitrogen trichloride from indoor swimming pools [67], chemical odours [68], and crude oil spills [69–71]. In the newspaper printing industry—which uses multiple chemicals including organic solvents, filler materials, and inks—a higher than expected incidence of chronic pharyngitis has been reported [72]. Sore throat is also a frequently-reported (72 %) symptom [73] in people with multiple chemical sensitivity syndrome.

The US World Trade Center disaster (9/11) resulted in uncharacterised hazardous exposures that are thought to be the cause of the upper airway inflammation seen in many of those exposed [74]. In 10,378 fire fighters, sore throat was rare before the disaster (3.2 % reported frequent sore throat), but was the most common respiratory symptom in the year afterwards (62.4 %), declining and plateauing thereafter (36 % in 2 years, and 37 % in 4 years) [75]. Along with cough, sore throat was considered to be the most sensitive initial indicator of respiratory insult [75].

Volcano eruptions emit sulphur dioxide and other gasses that may react with atmospheric components, and have been reported to cause sore throat. For example, sore/dry throat was significantly associated with volcanogenic sulphur dioxide and fine sulphate particles in Hawaii [76–78], and the hourly incidence of sore throat showed clear exposure–response relationships with post-eruptive SO_2_ concentrations [79].

Complaints of sore throat are frequent in communities with confined animal facilities, wastewater treatment plants, and biosolids recycling operations [80]. Sore throat was also significantly more prevalent in Greek solid waste collectors versus other workers [81]. Pig farming may be a particular cause of sore throat in nearby residents [82], with a 10 EU/mg increase in endotoxin being associated with increased log odds (0.10 ± 0.05) of sore throat [83]. Farmers are additionally exposed to pesticides, and this was attributed as the cause of pharyngitis in a study of farmers versus other workers in the United Arab Emirates [84].

These studies reveal that occupational and hazard-associated irritants, including particulates, chemicals, fumes and gasses, and endotoxin are well-documented causes of sore throat.

*Indoor air pollution* causes sick building syndrome, including sore throat. In the building assessment survey and evaluation (BASE) study, 7.1 % of workers in 41 large office buildings reported sore or dry throat [85]. The underlying source of pollutants is thought to be poorly maintained moisture-related heating, ventilating, and air-conditioning components such as cooling coils and humidification systems [86]. Thus, symptom prevalence is higher in buildings ventilated mechanically or with air-conditioning compared with naturally ventilated buildings [87, 88]. The specific pollutants responsible are not well characterised. Ozone brought into the building through the ventilation system [89], and its interaction with ventilation filters [90], has been implicated, as have particulates [91]. Damp and mouldy buildings also cause sore throats [92, 93].

*Temperature and humidity* affect mucus membranes, and have been linked with sore throat symptoms. Heated air causes nasal pain [94] and working regularly in a cold environment causes rhinitis and sore throat as well as changes in lung function [95]. Humidity is also important, with the overall intensity of sick building syndrome symptoms increasing when indoor air is not humidified [96]. Experimentally, cold dry air induces nasal inflammation [97, 98], and causes more nasal pain than humidified dry air [94]. However, a population-based study showed that both cold temperature and low humidity appear to independently increase the risk of sore throat [99].

## Management of non-infectious sore throat

There has been little systematic assessment of treatments for sore throat of non-infectious aetiology, and the field is hampered by a lack of objective outcomes, with most studies relying on subjective (self-reported) endpoints. By definition, studies of antibiotic treatment for sore throat are in infectious populations. Studies of non-antibiotic treatment are generally similarly confounded by the inclusion criteria ‘acute pharyngitis’ or ‘chronic pharyngitis’ which will likely be of mixed aetiology. A population with sore throat secondary to functional dysphonia might better reflect the non-infectious aetiologies, but such studies are few. Defined non-infectious study populations are, therefore, required. Many of the current data are unreliable for determining the efficacy of treatments for non-infectious sore throat, although some parallels can be drawn.

It is likely that the efficacy of non-antibiotic treatments that has been reported in acute (mostly infectious) sore throat might also be seen in non-infectious sore throat. This extrapolation is based on the assumption that simple analgesia and anti-inflammatory effects are likely to be effective whatever the aetiology, especially if neurogenic inflammation is involved (see below). A systematic review in acute sore throat found that treatments effective on early outcomes (<24 h) included steroids, non-steroidal anti-inflammatory drugs (NSAIDs), caffeine, and paracetamol, while paracetamol and NSAIDs also had a benefit on later (>24 h) outcomes [12]. These treatments might also work for non-infectious sore throat.

There have been some studies in chronic pharyngitis, but these are mostly small. Flurbiprofen has been shown to be effective [100], as have Chinese herbs used in combination with acupuncture [101]; Chinese herbs have been the subject of a systematic review [102]. Inhaled natural ether oils have also been used [103], although the composition is mostly unknown. A Russian study of 22 patients with aggravated chronic subatrophic pharyngitis against a background of acute respiratory viral infection showed that amylmetacresol/dichlorobenzyl alcohol lozenges (Strepsils^®^, Reckitt Benckiser Healthcare Ltd) resulted in a marked and durable reduction in pain symptoms, which was reported immediately after the first dose, and resulted in average pain scores three times lower than the control group by day 8 [104]. Although the aetiology of sore throat in these patients is not always well defined, several treatments show promise for the management of persistent sore throat.

Studies in defined non-infectious sore throat populations are few. Nimesulide and flurbiprofen were both found to be effective in a randomised study of 60 patients with non-infectious acute inflammation of the upper respiratory tract [105]. Experimentally, paracetamol in combination with caffeine reduces the nasal tonic pain elicited by dry air provocation, and caffeine enhances and prolongs the analgesia compared with control [106], supporting a role for analgesics in non-infectious sore throat. Patients with sore throat secondary to functional dysphonia may respond to treatment of the underlying cause with vocal hygiene, voice training, avoidance of irritants and allergens, and co-medications (e.g. proton pump inhibitors, anti-allergic drugs).

The most frequently studied non-infectious population is that of postoperative sore throat and owing to its high incidence, a great many studies have been conducted in this area. For example, postoperative sore throat can be prevented with inhaled fluticasone propionate [107], intravenous dexamethasone [108], and lidocaine [30]. Other remedies include ketamine gargle [109], topical NSAIDs including benzydamine [110], perioperative amylmetacresol/dichlorobenzyl alcohol lozenges (Strepsils^®^) [111], liquorice [112], and an azulene derivate from chamomile [113].

These studies in defined populations with non-infectious sore throat add to the data obtained in studies of less well-defined acute and chronic sore throat. Although there is currently a lack of rigorous trials of treatments for non-infectious sore throat, there are enough data to suggest there may be several effective strategies. The lack of a suitable preclinical model hampers efforts to identify effective new treatments.

## The mechanism of non-infectious sore throat

The treatment of non-infectious sore throat could be improved if the mechanisms were fully understood. However, the underlying pathophysiology, and components such as individual disposition and potential receptors and their interaction with irritants, are not yet well characterised. To complicate matters, the underlying mechanism may vary with aetiology. Inflammation is a logical consequence of infection of the upper respiratory tract. Inflammation has also been implicated in non-infectious aetiologies, with inflammatory cells and mediators isolated from the upper respiratory tract (for example [114, 115]).

Mechanical trauma and injury is almost certainly the precipitating factor in postoperative sore throat [25], as supported by the preventive efficacy of some lubricants [116]. The inflammatory response has been replicated in a swine model, with elevated interleukin (IL) 6 and polymorphonuclear leukocytes found in tracheal lavage fluid [117]. However, the precise aetiology and location of postoperative sore throat remains unknown [25]. Factors such as tracheal-tube size [118] and cuff design [119] have been implicated during intubation [26], and sore throat following the use of a laryngeal mask airway appears to be related to the technique of insertion [31] and intracuff pressure [26]. The problem is more common in older age groups, and is related to grade of difficulty of intubation, duration of surgery, and patient’s position during surgery [29], with all factors probably reflecting an increased risk of trauma.

The mechanism underlying sore throat in people with gastroesophageal reflux disorder is likely to be chemical, that is, due to refluxed gastric content, although indirect effects through vagal (sensory) mechanisms have also been implicated [42].

In rhinitis, pharyngeal irritation has been attributed to the lymphoid hypertrophy and prominence of adenoidal and tonsillar tissue, which results from chronic allergic inflammation of the upper airway [120], and kinins generated in nasal secretions have been implicated [121]. The mediators released after nasal challenge with allergens or cold dry air are remarkably similar, including histamine, leukotrienes, prostaglandin D2, and TAME-esterase [122]. The mechanism of cold air-induced rhinitis is well-characterised, being associated with nasal mast cell activation and sensory nerve stimulation (irritation) that generates a cholinergic reflex leading to rhinorrhoea [98]. It seems likely that the same mechanism may be responsible for cold-induced sore throat—a useful experimental model (see below).

Snoring is problematic in terms of identifying the mechanism because although sore throat may cause snoring, snoring may also cause sore throat [22]. The mechanism is likely to be mechanical and similar to that seen with dry air; sore throat is also associated with mouth breathing [7, 22], obviously due to drying out of the pharyngeal mucosa caused by unfiltered, unwarmed, non-humidified air passage. Snoring-induced vibrations have also been implicated in sore throat [22]. Similarly, it may be physical stress on the pharynx that causes sore throat following shouting and voice loading. Along with such mechanical mechanisms, emotional stress and stress-induced subjective factors may also play a role, perhaps in combination with muscle spasm. Excessive tension of the (para) laryngeal musculature (due to psychological and emotional factors, vocal misuse, or compensation for underlying problems such as infection) [123] may result in pain that could promote the persistence of lesions in the laryngeal and pharyngeal mucosa.

The drugs that frequently (2–40 %) cause sore throat or dry cough as a recognised adverse effect include ACE inhibitors, which increase pro-inflammatory mediators such as kinins, substance P, and prostaglandins [124]. For this unwanted drug effect genetic predisposition factors were found on corresponding receptors [124]. Switching from ACE inhibitors to angiotensine receptor blockers is the most accepted alternative to prevent elevated cytokines. The mechanism by which inhaled corticosteroids cause sore throat is less certain [37, 39]. Histologically, an inflammatory infiltrate has been reported in some patients [37] and locally-deposited steroid is likely to be a factor [125]. In contrast, oral steroids alleviate the pain of acute sore throat [13], probably due to their anti-inflammatory action (because of the risk of side effects they are not a routine therapeutic option in the long term). Chemotherapy and radiotherapy cause sore throat as a consequence of oropharyngeal mucositis [126].

These physico-chemical causes of sore throat, while not fully elucidated, at least have plausible putative mechanisms. The situation for many environmental insults is more complex. Throughout the literature, the term ‘irritants’ is used, but documented evidence of their identities or the mechanism by which they might irritate and inflame mucous membranes is scarce. For example, is it a physical interaction or do the irritants bind to receptors that trigger a deleterious response that manifests symptomatically as sore throat? Identification of such receptors would certainly go a long way to establishing the mechanism and providing a therapeutic target.

Tobacco smoke is an often cited irritant [14]. It irritates the stratified squamous epithelium of the oropharyngeal mucosa [127] causing damage and reduced mucociliary clearance, and impairs the immune response [128]. Nicotine binds to the nicotinic acetylcholine receptor on sensory neurons [129, 130], and this is one of the proposed mechanisms for irritation and pain caused by cigarette smoke. However, chronic pain and an acute analgesic effect of nicotine attenuated by desensitisation and/or upregulation of the receptor on chronic exposure have also been suggested [131]. Smoking also alters the resident flora [132] predisposing to infection. Conversely, abstaining from smoking for even one week causes a significant increase in reports of sore throat (*P* = 0.049) and other cold symptoms [133]. This is perhaps due to reduced salivary immunoglobulin A [134]. Furthermore, the reason for this unexpected observation might be rebound sensitisation of (nicotinic acetylcholine) receptors.

Ozone has an inflammatory effect on both the upper and lower respiratory tract, characterised by polymorph infiltration and release of pro-inflammatory cytokines [135–137], although the specifics relating to sore throat are less certain. The underlying mechanism is proposed to include a direct effect of ozone on respiratory tract cell membranes and fluid, with lipid ozonation products activating specific lipases, triggering the release of endogenous mediators of inflammation [138, 139] such as prostaglandin E, IL8, thromboxane B2 and calcitonin gene-related peptide.

A straightforward chemical mechanism may underlie the irritant effect of some environmental pollutants. For example, in the presence of metal catalysts and moist air, SO_2_ forms sulphuric acid and ammonium sulphate [45]. Other pollutants probably include NO_2_, and boron oxide and boron acid. Sore throat in response to chemical odours [68] could be due to irritation by the odorants themselves or co-pollutants, or a learned aversion [140] which is well-established for odorants.

Particulate matter, which contains both organic and inorganic constituents, is associated with airway inflammation, apoptosis, genotoxicity, and oxidative stress [141–143]. Cement dust induces atrophic and hypertrophic changes in nasal and pharyngeal mucosa and chronic exfoliative bronchitis in animal models [144]. Much of the extensive research in the particulates field has been conducted in vitro, but humans exposed to fuel oil ash have evidence of inflammation, with increased IL8 and polymorphs in nasal lavage fluid [114]. Induced sputum studies of fire fighters 10 months after the World trade centre disaster found levels of neutrophils and eosinophils increased in relation to the intensity of dust exposure [115].

Neurogenic inflammation may be responsible for the airway response to particulate matter and other environmental irritants such as ozone or sulphur dioxide [145, 146]. The irritants are proposed to interact with transient receptor potential (TRP) channels. These ion channels are found on sensory nerve fibres as well as immune and non-immune cells in the respiratory tract, and their activation results in the release of neuropeptides, such as calcitonin gene-related peptide and substance P, that trigger inflammation [147]. TRP channels are normally involved in detection of the thermal, mechanical, and chemical stimuli that are essential for survival [148].

Activated and sensitised TRP channels, particularly TRPV1 and TRPA1, have been implicated in chronic airways diseases (chronic obstructive pulmonary disease and asthma) and the respiratory response to environmental irritants [148, 149]. TRPV1 is activated by the potent respiratory irritant capsaicin, but also by extracellular protons (generated during tissue acidosis and ischaemia) and heat [149], and is also directly sensitised by nicotine [148]. TRPA1 is activated by chlorine, reactive oxygen species, and noxious constituents of smoke and smog [149], as well as ginger and bradykinin [150]. It appears that reactive respiratory irritants may activate TRPA1 by covalent modification, with glutathione depletion perhaps prolonging the effect [149]. The cumulative effect may result in a reduced threshold for TRPA1 activation, such that even low levels of smog or indoor pollutants elicit a response [149].

Along with this direct activation of TRP channels by irritants, the pain associated with inflammation may also be mediated by TRP channels. TRPV1 and TRPA1 are both activated and sensitised through inflammatory receptor pathways [149]. Hence, when pro-inflammatory mediators (such as histamine, prostaglandins, cysteinyl leucotrienes, proteases, nerve growth factor, and bradykinin) bind to their respective receptors on sensory neurons, this indirectly results in TRP activation [149] via the endogenous activating lipids produced by the phospholipase C and phospholipase A2 pathways [148].

The field of TRP channel research is rapidly expanding, as it becomes clear that these ion channels may be involved in a vast range of diseases and chronic pain situations. Because pain can be the result of overly sensitised TRP channels, it follows that TRP desensitisation using agonists might provide relief [151]. This is supported by animal models, where capsaicin desensitises the acute and inflammatory airway response to a variety of noxious stimuli [149].

With the sensory role of TRPs in detecting a wide range of thermal, mechanical, and chemical stimuli, TRP channel activation and sensitisation—and the consequent triggering of inflammation—potentially accounts for many of the aetiologies of non-infectious sore throat, providing the possibility of interactions of TRP channels and targets for intervention. Along with TRPV1 and TRPA1, other possible mechanisms and targets for intervention include: the somatosensory TRP channels, TRPM8, TRPV2 and TRPV4 [152]; acid-sensing ion channel in the airway, which detects acidity and toxic gasses [146]; and the neuronal nicotinic acetylcholine-receptor [129, 130].

## Experimental models of non-infectious sore throat

In order to fully elucidate the pathophysiology of non-infectious sore throat, as well as assess the efficacy of any potential treatments, models with defined conditions and objective endpoints are required.

Many of the currently available models are not specific for sore throat, and use subjective endpoints. Since the sensation of pain is so subjective, asking patients to rate its severity can often be the most appropriate endpoint. Models for the study of non-allergic rhinitis and cough, but not specifically sore throat, include challenge with methacholine, histamine, capsaicin [153], mechanical pressure [154], and citric acid [155, 156]. In contrast, nasal provocation with bradykinin induces sore throat as well as rhinitis [121]. The sore throat pain model [157, 158], by definition, provides specificity. However, it requires patients to rate their throat pain on a visual analogue rating scale and, as such, the endpoint is subjective.

There are some more specific animal models with objective endpoints. Pharyngeal inflammation was induced in a rat model by applying capsaicin directly to the pharyngeal mucosa [159]. Intubation has been used to induce pharyngeal inflammation in a swine model [117] and laryngeal inflammation in a rat model [160], with analysis of biomarkers. Well-defined human models of pharyngeal irritation with objective endpoints are generally lacking. One that is in research use is the Tonsilliopharyngeal assessment (TPA) scale which is based on a clinical examination of the throat [158], although this still depends on the clinician’s judgement. Currently, any correlation between subjective and objective endpoints is therefore untested.

In humans, biomarkers, such as immune cells and inflammatory mediators, are often experimentally measured in epidemiological studies, providing an objective marker of airway inflammation. However, unless obtained from a biopsy (which is too invasive for routine use), these are not necessarily specific to the pharyngeal mucosa. For example, analysis of bronchiolar lavage fluid reflects inflammation occurring in the lungs but is relatively invasive to perform, so not in common use. Tracheal lavage and induced sputum techniques also sample mostly the lower respiratory tract. Nasal lavage, which samples the upper respiratory tract, gets closer to the pharyngeal mucosa, but is still relatively specific to the nasal epithelium. In contrast, pharyngeal lavage [161] is site specific.

In humans, nasal challenge with cold dry air has been used to experimentally replicate cold air-induced rhinitis [97, 98, 153] and can be combined with the objective analysis of biomarkers of nasal inflammation.

## A new model of pharyngeal inflammation in humans

The lack of suitable models for the study of non-infectious sore throat has hampered progress in elucidating mechanisms and identifying treatments. A new model has been developed using cold dry air (CDA) to experimentally induce controlled inflammation in the pharynx of healthy volunteers. The inflammation is then quantified by measuring inflammatory mediators in pharyngeal lavage fluid [unpublished].

The model is a promising tool for future research because it induces reversible pharyngeal irritation, in controlled conditions, and is amenable to a crossover study design. It may provide a valuable research tool and an objective way of measuring the therapeutic potential of treatments. Furthermore, this procedure may also allow for research in occupational and environmental medicine where exposure limits for single potential irritants exist but interactions are not yet well established. In this way, a CDA stimulus may be combined with one or two potential irritants in order to generate comparable results in crossover study designs.

## Conclusions

Despite how common non-infectious sore throat is, little is understood about how it is caused by such a wide variety of physico-chemical and environmental factors. It is possible that in many cases the cause of persistent non-infectious sore throat involves a combination of physico-chemical and environmental factors. Inflammatory processes (of various mechanisms, including neurogenic inflammation) appear to underlie sore throat of non-infectious aetiologies, and in many cases this may be the source of the pain. Cytokine receptors and TRP channels seem to be involved in the transduction pathways. Inflammation also underlies infectious sore throat, so many of the therapeutic options may be also appropriate for non-infectious sore throat. Indeed, although limited, the literature suggests that appropriate treatment options for non-infectious sore throat may include drug therapy. However, the lack of suitable human experimental models hampers the identification of drug therapy (for example topical TRP antagonists). A new model using cold dry air and pharyngeal lavage may provide a useful tool for the study of mechanisms—including receptor interactions—and treatment of non-infectious sore throat.
